# Five steps in leukocyte extravasation in the microcirculation by chemoattractants

**DOI:** 10.1155/S0962935192000619

**Published:** 1992

**Authors:** T. Oda, M. Katori, K. Hatanaka, S. Yamashina

**Affiliations:** 1 Department of Pharmacology Kitasato University School of Medicine Kanagawa Sagamihara 228 Japan; 2 Department of Anatomy Kitasato University School of Medicine Kanagawa Sagamihara 228 Japan; 3 Department of Biology Faculty of General Education Yamagata University Yamagata 990 Japan

## Abstract

For in vivo study of the phenomena observed in vitro, PMN (polymorphonuclear leukocyte) extravasation was analysed quantitatively in the microcirculation of the hamster cheek pouch using a video system. Topical application of leukotriene B_4_ or N-formyl-methionylleucyl- phenylalanine increased dose dependently the number of PMNs adhering to the venules. Eighty to 90% of the adhering PMNs disappeared from the vascular lumen into the venular wall within 10-12 rain after the adhesion. After PMNs had passed through the endothelial cell layer, they remained in the venular wall for more than 30 min after application of the chemoattractants and appeared in the extravascular space. Thus, the process could be divided into five steps: (1) rolling and (2) adhesion to the endothelium, (3) passage through the endothelial layer (4) remaining in the venular wall, and (5) passage through the basement membrane.


Mediators of Inflammation 1, 403–409 (1992)

### Five steps in leukocyte extravasation in the microcirculation by chemoattractants

T. Oda,*[,1], M. Katori,[1CA]

K. Hatanaka[1] and S. Yamashina[2]

[1]Department of Pharmacology and [2]Anatomy, Kitasato University School of Medicine, Sagamihara, Kanagawa 228, Japan,
*Present Address: Department of Biology, Faculty of General Education, Yamagata University, Yamagata 990, Japan

[CA] Corresponding Author

**FOR *in vivo* study of the phenomena observed *in vitro*, PMN (polymorphonuclear leukocyte) extravasation was analysed quantitatively in the microcirculation of the hamster cheek pouch using a video system. Topical application of leukotriene $B_4$ or *N*-formyl-methionyl-leucyl-phenylalanine increased dose dependently the number of PMNs adhering to the venules. Eighty to 90% of the adhering PMNs disappeared from the vascular lumen into the venular wall within 10–12 min after the adhesion. After PMNs had passed through the endothelial cell layer, they remained in the venular wall for more than 30 min after application of the chemoattractants and appeared in the extravascular space. Thus, the process could be divided into five steps: (1) rolling and (2) adhesion to the endothelium, (3) passage through the endothelial layer (4) remaining in the venular wall, and (5) passage through the basement membrane.**

**Key words:** fMLP, $LTB_4$, Neutrophil adhesion, Neutrophil extravasation, Vascular basement membrane

### Introduction

Transmigration of polymorphonuclear leukocytes (PMNs) from the circulation to the extravascular space is one of the cardinal signs of the acute inflammatory responses. At the microcirculatory level, PMNs have been described as rolling along the venular wall[1,2] and then adhering to the inner surface of the endothelial cells.[1,3–5] Studies by electron micrography have also indicated that PMNs penetrate the vessel wall between endothelial cells.[6–10] However, past observations, made *in vivo* at the microcirculatory level, of the process of transmigration of PMNs have been mainly rather descriptive, and not quantitative.

The initial report from *in vitro* experiments that incubation of interleukin-1 with cultured endothelial cells from the human umbilical vein increased their adhesivity for neutrophils[11] stimulated studies on adhesion molecules. Since then, many findings on adhesion molecules of neutrophils and endothelial cells have been accumulated using cultured endothelial cells and isolated neutrophils.[12,13] Two intercellular adhesion molecules, ICAM-1 (intercellular adhesion molecules-1)[14] and ICAM-2[15] have been identified on endothelial cells in relation to neutrophil adhesion to these cells.

Simultaneously, studies of patients who show repetitive infection and lack of PMN migration[16] have revealed congenital deficiencies of a family of surface glycoproteins of leukocytes, which have now been identified as adhesion molecules of the integrin superfamily (CD11/CD18).[17]

In spite of the extensive efforts made in examining adhesion molecules *in vitro*, in order to understand properly the phenomenon of neutrophil extravasation, which occurs *in vivo* at the microcirculatory level, confirmation *in vivo* of the *in vitro* findings is essential. Also, in order to discuss PMN transmigration at the molecular level *in vivo*, quantitative analyses are required.

We reported a quantitative analysis of the rolling and adhesion of PMNs induced by leukotriene (LT) $B_4$ and *N*-formyl-methionyl-leucyl-phenylalanine (fMLP) in the microcirculation of the hamster cheek pouch.[5] The behaviour of PMNs in the vascular lumen was quantified from changes in intensity of images transmitted from a monochrome television (TV) camera to a TV monitor screen, by using a photocell placed on this screen, but the subsequent steps of PMN extravasation remain to be analysed.

The present experiments aim to give a quantitative analysis of the entire process of the transmigration of PMNs from the bloodstream to the perivascular space at microcirculatory levels in the cheek pouch of anaesthetized hamsters.

### Materials and Methods

*Preparation of hamster cheek pouch:* The hamster cheek pouch preparations were set up as previously described.[3,5,18] Tracheal intubation ensured spontaneous respiration. The male golden hamsters (*Mesocricetus auratus*, 120–150 g, 10–16 weeks old) were anaesthetized with pentobarbital (Nembutal®,

© 1992 Rapid Communications of Oxford Ltd

Abbot Lab, Chicago, IL, USA) (60 mg/kg, i.p.). The cheek pouch was everted, cut longitudinally, and extended. The avascular connective tissue was elaborately dissected away to expose the microvasculature of the mucous layer. The thin mucous membrane tissue was spread in a plastic chamber (9 ml) and superfused at 5 ml/min with warmed Tyrode's solution, which was maintained at 37°C. Body temperature was maintained with a warming pad positioned directly beneath the hamster and maintained at 37°C.

*Microscopic observation and recording of leukocyte behaviour:* The microvasculature of the hamster cheek pouch was observed with transillumination under a microscope (BHA, Olympus Optical Co. Ltd, Tokyo), using a water immersion lens ( × 40) and × 10 eyepieces (Olympus Optical Co. Ltd, Tokyo). Images of the microcirculation were projected into a TV monitor screen via a monochrone TV camera (CTC 2600, Ikegami Tsushinki, Tokyo) mounted at the top of a triocular microscope. The leukocyte responses in each experiment were usually recorded with a videotape recoder (VO-5850, Sony Co., Tokyo) and replayed after the experiments. The rolling behaviour, adhesion to the vessel walls, and migration to the extravascular space, of individual leukocytes were clearly visible on the screen. The observation and experimental procedures were performed only once for each animal. The behaviour of individual leukocytes in a post-capillary venule of 13–15 $\mu$m diameter were observed over a 110 $\mu$m length along a venule. During the 30 min equilibration period, marginating leukocytes could be visualized inside the venules as they rolled along the endothelium at a lower velocity than the bulk flow.

*Chemotactic agents:* $LTB_4$ (Paesel, Gmbh and Co, Frankfurt) and fMLP (Peptide Institute, Minoh, Osaka) were used as chemoattractants. Stock solutions of $LTB_4$ (30 $\mu$M in absolute ethanol) or fMLP (1 mM in saline) were kept frozen at $-70$°C and diluted to appropriate concentrations with Tyrode's solution immediately before use.

*Application of $LTB_4$ and fMLP:* The superfusion of the cheek pouch with Tyrode's solution was stopped during the experiments. $LTB_4$ or fMLP solution was applied to the microvasculature at the observation sites with a 50 $\mu$l micropipette.

*Electron microscopy:* Before and 30 min after the application of $LTB_4$, parts of the thin preparation of the hamster cheek pouch used for examination of the microcirculation were fixed in 2.5% glutaraldehyde and 1% osmium tetroxide. Ultrathin sections were cut on a LKB ultramicrotome and were stained with uranyl acetate and lead citrate, and observed by a JEM-1200EX electron microscope (JEOL Ltd, Tokyo).

### Results

*Rolling:* In the absence of chemoattractants, a few PMNs could be individually visualized as bright white cells against the dark background of the blood stream, since they rolled slowly on the endothelial wall. Although the number of rolling PMNs was quite variable from preparation to preparation, the majority of the rolling PMNs moved in a jerky fashion, then came to rest suddenly for short, but variable periods of time (3–30 s), and finally returned to the bloodstream (Fig. 1). The behaviour of a PMN moving slowly along the endothelial surface and stopping at the same site for less than one minute was designated as 'rolling'. Some PMNs rested at the same site for longer periods, but eventually they moved again (Fig. 2).

On the application of a chemoattractant (either $LTB_4$ or fMLP) the number of rolling PMNs was kept constantly low and was not increased (Fig. 2),

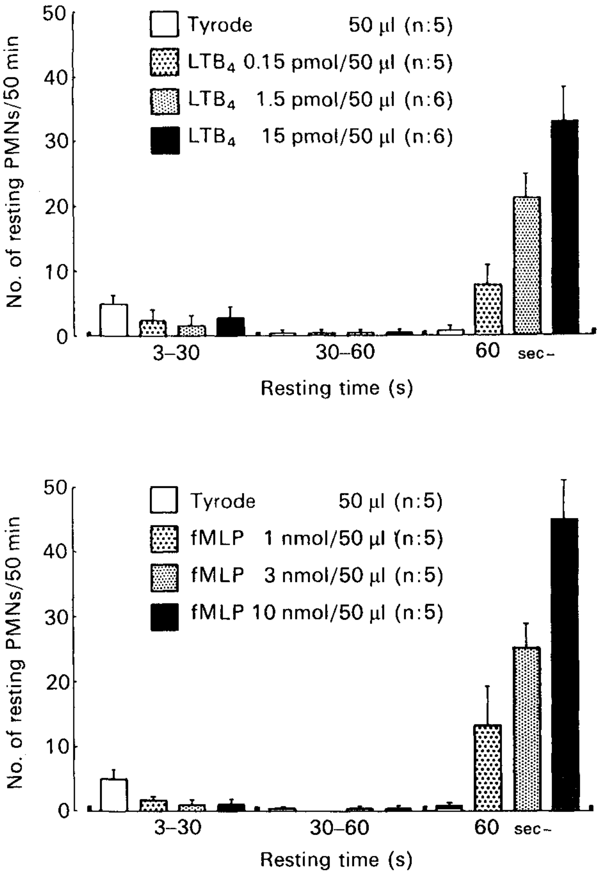

FIG. 1. Times for which PMNs remained at the same site in a venule after topical application of $LTB_4$ and fMLP. The abscissa indicates the ranges of time spent by the same PMNs at the same site on the inner surface of the venules. The ordinate indicates the cumulative numbers of PMNs remaining for each time range, counted in a 110 $\mu$m length along a venule on the monitor screen for 50 min after the application of $LTB_4$ or fMLP. Values indicate the mean ($\pm$SE) from five to six animals.

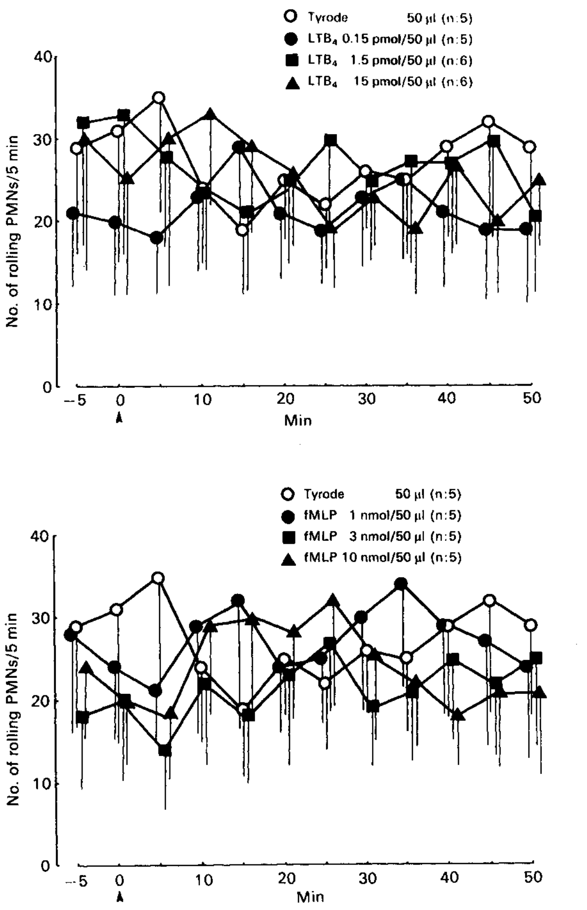

FIG. 2. Changes in the numbers of rolling PMNs after $LTB_4$ and fMLP application. The abscissa indicates the time lapse after the application of $LTB_4$ or fMLP (arrowhead, 0). The ordinate indicates the numbers of PMNs counted in a 110 μm length along a venule on the monitor screen, recorded for 50 min after application of $LTB_4$ or fMLP. Values indicate the means (±SE) obtained from the respective number of animals (*n*).

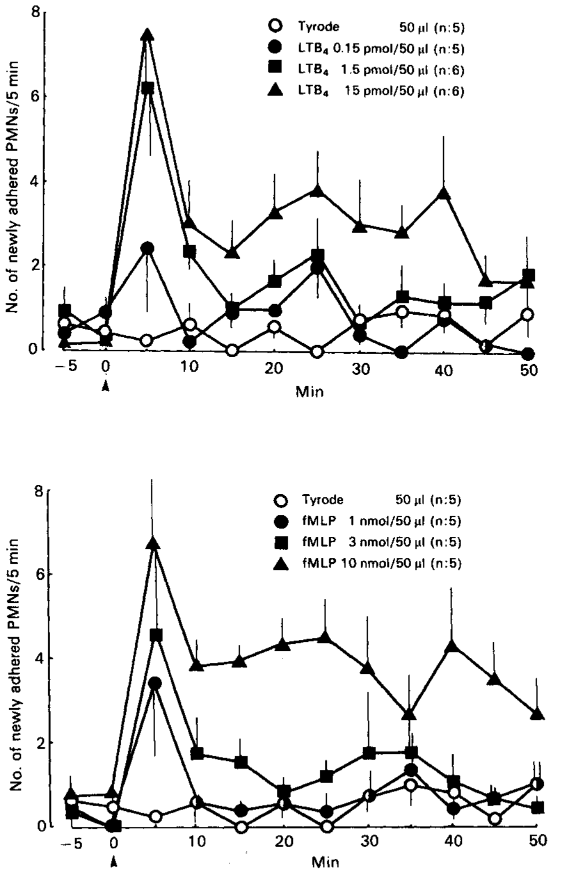

FIG. 3. Changes in the numbers of adhering PMNs after $LTB_4$ and fMLP. The abscissa indicates the time lapse after $LTB_4$ or fMLP application (arrowhead, 0). The ordinate indicates the numbers of PMNs newly adhering to a 110 μm length along a venule on the monitor screen, recorded for 50 min after $LTB_4$ or fMLP application. Values indicate the means (±SE) obtained from the respective numbers of animals (*n*).

whereas the number of PMNs remaining for longer than one minute at the same site was markedly increased dose dependently (Fig. 1). The behaviour of a PMN remaining at the same site for one minute or longer, was designated as 'adhesion'.

*Adhesion:* Figure 3 indicates the changes in the number of adhering PMNs in a 5 min period upon topical application of a chemoattractant. In absence of chemoattractants, the number of adhering PMNs was constantly low. Immediately after the application of $LTB_4$ or fMLP, adhesion was clearly induced. The number of adhering PMNs was increased dose dependently by the application of either $LTB_4$ or fMLP, and then the effect slowly waned. The cumulative numbers of adhering PMNs over a period of 50 min after the application of 0.15, 1.5, and 15 pmol/50 μl of $LTB_4$ were 7.8 ± 3.1 ($n = 5$), 21.0 ± 3.9 ($n = 6$), 31.8 ± 6.4 ($n = 6$) (mean ± SE), respectively and those after 1, 3 and 10 nmol/50 μl of fMLP were 13.3 ± 6.2 ($n = 5$), 25.7 ± 3.5 ($n = 5$) and 45.0 ± 6.0 ($n = 5$), respectively.

*Passage through the endothelial cell layer:* Observation of PMNs on the videotapes revealed that those PMNs that were adhering to the venular wall had a large area of contact with it (Fig. 4B). This contact area was then seen to diminish as the cells became thinner and 'taller' (Fig. 4C), and then to decrease their 'height' gradually (Fig. 4D and E), until they finally disappeared from the vascular lumen. The

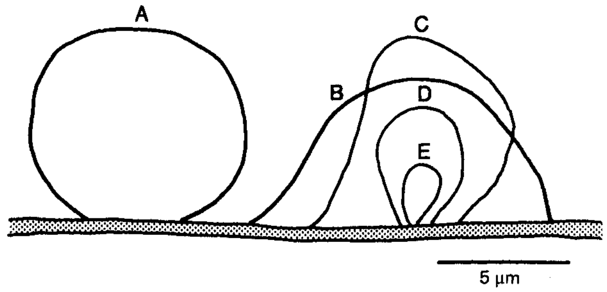

FIG. 4. Schematic illustration of the shape of one PMN that was rolling and then adhered to the surface of a venule and disappeared from the vascular lumen after the application of $LTB_4$. This illustration was reconstructed from a series of frames on the videotape after the application of $LTB_4$ (15 pmol/50 μl). (A) Rolling PMN, 3 s before adhesion. (B) The onset of adhesion, 0 sec. (C) 1 min and 10 s after adhesion. (D) 4 min and 40 s after adhesion. (E) 5 min and 10 s after adhesion.

**Table 1.** Time from adhesion of PMNs to disappearance from the vascular lumen after the application of $LTB_4$ and fMLP

| Treatment | Numbers of animals used | Numbers of PMNs observed | Time to disappearance mean ± SE (min) |
|---|---|---|---|
| $LTB_4$ (pmol/50 μl) | | | |
| 0.15 | 5 | 10 | 9.5 ± 1.9 |
| 1.5 | 6 | 30 | 11.4 ± 1.1 |
| 15 | 6 | 30 | 10.0 ± 0.9 |
| fMLP (nmol/50 μl) | | | |
| 1 | 5 | 10 | 9.2 ± 2.1 |
| 3 | 5 | 20 | 10.2 ± 1.0 |
| 10 | 5 | 25 | 12.0 ± 1.3 |

average time from the onset of adhesion to disappearance from the vascular lumen was about 10 min, irrespective of the dose of $LTB_4$ or fMLP (Table 1). The shortest time recorded was 3 min and the longest was 25 min.

Figure 5 indicates that 80–90% of the PMNs, which had adhered to the venular wall, disappeared into it irrespective of the dosage of $LTB_4$ (1.5 to 15 pmol/50 μl) or fMLP (1 to 10 nmol/50 μl), and never returned to the bloodstream.

*Presence in venular wall:* Subsequent to passage through the endothelial cell layer, PMNs remained for longer than 30 min in the venular wall between the endothelial cell layer and the basement membrane of the pericytes/endothelial cells, as shown in the electron micrograph in Fig. 6. Parts of the vascular wall were seen on the TV monitor screen to become thicker, possibly as a result of the PMNs present in the wall.

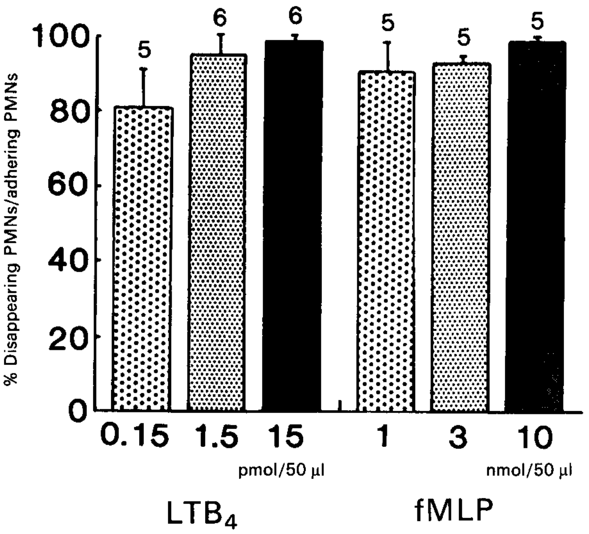

FIG. 5. Number of PMNs that disappeared from the vascular lumen as percentages of those of PMNs that adhered after $LTB_4$ and fMLP were applied. The abscissa indicates the concentrations of $LTB_4$ or fMLP. The ordinate indicates the numbers of PMNs that disappeared from the vascular lumen expressed as percentages of PMNs that adhered, as recorded for 50 min after the application of $LTB_4$ or fMLP. Values indicate the means (±SE) from the numbers of animals indicated at the top of the respective columns.

*Migration into extravascular spaces:* The number of PMNs in the extravascular space, observed up to 90 min after the application of $LTB_4$ or fMLP, increased with time (Fig. 7). When observed on the monitor screen during its migration into an extravascular space, a PMN would extend the process through an exit in the vascular wall into the extravascular space, and this was followed by amoeboid movement of the whole cell into the interstitial space.

The time taken for a PMN to enter the extravascular space completely ranged from 11 to 49 min, with a mean time of 25 to 34 min (Table 2).

Histological examination of the hamster cheek pouch revealed that the migrating cells were almost exclusively of the PMN type or neutrophils.

### Discussion

The photocell method of quantitative analysis of PMN behaviour, described in the previous paper,[5] is useful for when PMNs are rolling and adhering to the inner wall of the venules. However, the method is of no use after penetration of the endothelial cell layers by the PMNs. In the present experiments, the number of PMNs was counted

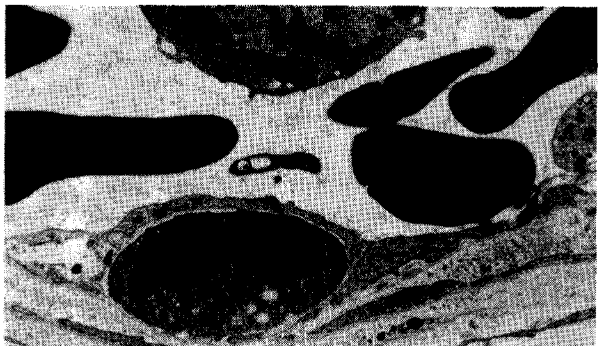

FIG. 6. An electron micrograph of a postcapillary venule from the hamster cheek pouch during PMN extravasation. A PMN remains between the endothelial layer and the basement membrane of the venular wall for 30 min after application of $LTB_4$ (15 pmol/50 μl). A scale at the left bottom indicates 1 μm. ×6500.

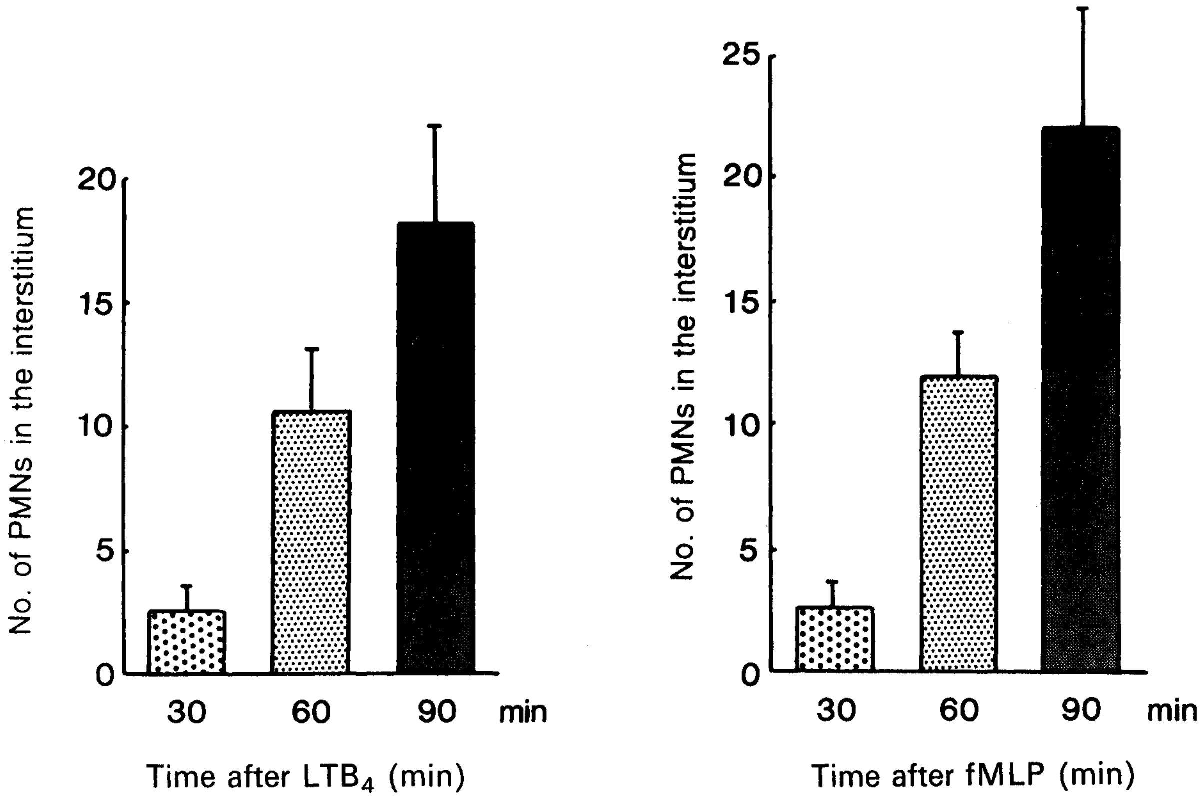

FIG. 7. Cumulative numbers of PMNs appearing in the extravascular space after $LTB_4$ or fMLP application. The abscissa indicates the time lapse after the application of $LTB_4$ or fMLP. The ordinate indicates the total numbers of PMNs in the perivascular space at given times after the application of $LTB_4$ (15 pmol/50 μl) or fMLP (10 nmol/50 μl). Values indicate the means (±SE) from five to six animals.

with the naked eye directly on the TV monitor screen during repeated replays of videotapes. This was not an elegant technique, but adequate for quantifying the behaviour of PMNs during extravasation.

In the absence of chemoattractants, only a few PMNs were visible rolling or remaining on the inner wall of the venules. The period of time for remaining at the inner wall was 3–30 s, and then the PMNs returned to the bloodstream. Topical application of $LTB_4$ or fMLP on the surface of the microvasculature did not change the number of rolling PMNs, but resulted in an increase in the number that remained adhering at the same site for at least 60 s (Fig. 1). Thus, the designation of rolling, in which PMNs were stationary for less than 1 min at one site, and that of adhesion, in which they remained at the same site for 1 min or longer, are reasonable. A small number of rolling PMNs were present in the venules before the application of chemoattractants, but this may be attributable to the manipulation of the cheek pouch during the extensive dissection during setting up the preparation and this manipulation may have caused the release of endogenous agents. The rolling behaviour of PMNs may be related to activation of the selectin family.[19]

The topical application of a chemoattractant

**Table 2.** Time from disappearance of PMNs from the vascular lumen to appearance in the perivascular space after $LTB_4$ and fMLP application

| Treatment | Numbers of animals used | Numbers of PMNs observed | Time to disappearance mean ± S.E. (min) |
|---|---|---|---|
| $LTB_4$ (pmol/50 μl) | | | |
| 0.15 | 5 | 10 | 32.4 ± 3.0 |
| 1.5 | 6 | 30 | 25.3 ± 1.4 |
| 15 | 6 | 30 | 31.6 ± 1.9 |
| fMLP (nmol/50 μl) | | | |
| 1 | 5 | 10 | 29.8 ± 2.7 |
| 3 | 5 | 20 | 34.0 ± 2.0 |
| 10 | 5 | 25 | 31.8 ± 1.9 |

($LTB_4$ or fMLP) increased the cumulative numbers of adherent PMNs, in accordance with the concentration used (Fig. 3). This result confirms those of the previous paper.[5] As the adhering and migrating cells were almost exclusively of the PMN type or neutrophils, as shown by histological examination, it can be concluded that $LTB_4$ or fMLP induced extravasation of neutrophils.

After *in vitro* experiments, the potency of $LTB_4$ was reported to be equipotent to that of $C_{5a}$ in chemotaxis,[20] to that of $C_{5a}$-des-Arg or fMLP in chemokinesis and chemotaxis,[21] and weaker than fMLP in release of the lysosomal enzymes from human PMN in the presence of cytochalacin B.[22–25] In the present *in vivo* experiments, the concentration of fMLP for induction of PMN adhesion was 1000 times higher than those of $LTB_4$. This is in agreement with the previous report.[5] The reasons for the discrepancy between *in vitro* and *in vivo* experiments are not known, but hamster PMNs may be less sensitive to fMLP, and, as has already been reported,[5] the effect of fMLP on adhesion of PMNs to the inner wall of venules is indirect in the hamster, since it is completely inhibited by a 5-lipoxygenase inhibitor. Despite the difference in potency, $LTB_4$ and fMLP share similar effects on the process of the extravasation of PMNs in the microcirculation of the hamster cheek pouch.

Adhesion of PMNs was observed only in the venules. The increased adhesion of leukocytes is primarily due to elevated adhesive forces and not the result of decrease in dispersive forces, e.g., wall shear stress.[26] Induction of PMN adhesion to the venular endothelium was attributable to changes in the membrane of the PMNs and not to that of the endothelial cells, as was indicated in the previous paper,[5] in which selective application of a minute amount of $LTB_4$ with a glass capillary pipette close to the capillary wall induced adhesion in the venules downstream, whereas injection close to the venular wall did not cause the adhesion of PMNs to it, but induced adhesion in the larger venules further downstream. High-affinity receptors for fMLP are reported to be present on the surfaces of leukocytes[27] and of cultured endothelial cells.[28] Thus, as indicated by *in vitro* experiments. Mac-1 (CD11b/CD18) and/or LAF-1 (CD11a/CD18) may be adhesion molecules of neutrophils, which are activated by $LTB_4$ or fMLP and bound to the counter-receptor ICAM-1 on the surface of endothelial cells.[12] In our preliminary experiments *in vivo*, this adhesion in the hamster check pouch was blocked by an antibody against rat CD18 or ICAM-1 (data to be published).

The present quantitative study revealed that almost all (80–90%) of the PMNs that had adhered to the venular walls, disappeared from the vascular lumen through the endothelial cell layer and did not return to the bloodstream. Thus, the adhesion of PMNs to the endothelial cells is the pivotal step in the process of PMN extravasation.

The disappearance of a PMN from the vascular lumen into the venular wall took about 10 min. This movement of PMNs must require detachment or inactivation of Mac-1 and LFA-1 with crosstalk to other adhesion molecules. The disappearance of PMNs from the vascular lumen probably takes place by passage through the endothelial cell junctions, as indicated by electron micrographs.[6–10]

PMNs that had passed through the endothelial cell layer did not immediately appear in the perivascular space, but remained in the spaces between the endothelial cells and the basement membrane of the pericytes/endothelial cells. This observation confirmed the findings of previous reports.[6,10,29] An important finding of the present study was that nearly 30 min elapsed before the appearance of PMNs in the extravascular space from disappearance from the vascular lumen (Table 2). PMNs may split the basement membrane with collagenase/gelatinase, since the last step was inhibited by a selective inhibitor of metalloproteinases (data published separately). Thus, 30 min may be the time required for PMNs to prepare metalloproteinases for action. Furthermore, this last step of the passage of PMNs through the basement membrane was inhibited by pretreatment of the animals with dexamethasone.[30,31]

The results presented above indicate that PMN extravasation by chemoattractants is not a simple phenomenon, but a serial process in which a variety of adhesion molecules on the surface of PMNs, endothelial cells and other components of the venular wall may be involved. The process can be divided into five steps: (1) rolling on the endothelium, (2) adhesion to the endothelium, (3) passage through the endothelial layer, (4) presence between the endothelial cells and the basement membrane within the venular wall, and (5) passage through the basement membrane into the extravascular space. Quantitative analysis of the extravasation of PMNs is necessary to clarify precisely the underlying mechanism, particularly at the molecular level. Individual steps may be targeted for development of new drugs, which inhibit PMN migration.

### References

1. Atherton A, Born GBR. Quantitative investigations of the adhesiveness of circulating polymorphonuclear leukocytes to blood vessel walls. *J Physiol* 1972; **222**: 447–474.
2. Clark ER, Clark EL. Observations on changes in blood vascular endothelium in the living animal. *Am J Anat* 1935; **57**: 385–438.
3. Björk J, Arfors KE, Hedqvist P, Dahlén SE, Lindgren JA. $LTB_4$ causes leukocyte emigration from the postcapillary venule in the hamster cheek pouch. *Microcirculation* 1982; **2**: 271–281.
4. Lindbom L, Hedqvist P, Dahlén S-E, Lindgren JA, Arfors K-E. Leukotriene $B_4$ induced extravasation and migration of polymorphonuclear leukocytes *in vivo*. *Acta Physiol Scand* 1982; **116**: 105–108.

5. Nagai K, Katori M. Possible changes in the leukocyte membrane as a mechanism of leukocyte adhesion to the venular walls induced by leukotriene $B_4$ and fMLP in the microvasculature of the hamster cheek pouch. *Int J Microcirc Clin Exp* 1988; **7**: 305–314.
6. Faustman PM, Dermietzel R. Extravasation of polymorphonuclear leukocytes from the cerebral microvasculature. *Cell Tissue Res* 1985; **242**: 399–407.
7. Hurley JV, Xeros N. Electron microscopic observations on the emigration of leukocytes. *Aust J Exp Biol* 1961; **39**: 609–624.
8. Marchesi VT. The site of leukocyte emigration during inflammation. *Q J Exp Physiol* 1961; **46**: 115–118.
9. Shaw JO. Leukocytes in chemotactic-fragment-induced lung inflammation. Vascular emigration and alveolar surface migration. *Am J Pathol* 1980; **101**: 283–302.
10. Thureson-Klein A, Hedqvist P, Lindbom L. Ultrastructure of polymorphonuclear leukocytes in postcapillary venules after exposure to leukotriene $B_4$ *in vivo*. *Acta Physiol Scand* 1984; **122**: 221–224.
11. Bevilacqua MP, Pober JS, Wheeler ME, Cotran RS, Gimbrone MA, Jr. Interleukin-1 acts on cultured human vascular endothelium to increase the adhesion of polymorphonuclear leukocytes, monocytes, and related leukocyte cell lines. *J Clin Invest* 1985; **76**: 2003–2011.
12. Carlos TM, Harlan JM. Membrane proteins involved in phagocyte adherence to endothelium. In: Moller G, ed. *Immunol Review*. Copenhagen: Munksgaard, 1990 (No. 144); 5–28.
13. Larson RS, Springer TA. Structure and function of leukocyte integrins. In: Moller G, ed. *Immunol Review*. Copenhagen: Munksgaard 1990 (No. 144); 181–217.
14. Rothlein R, Dustin ML, Marlin SD, Springer TA. A human intercellular adhesion molecule (ICAM-1) distinct from LFA-1. *J Immunol* 1986; **137**: 1270–1274.
15. Staunton DE, Dustin ML, Springer TA. Functional cloning of ICAM-2, a cell adhesion ligand for LFA-1 homologous to ICAM-1. *Nature* 1989; **339**: 61–64.
16. Harlan JM. Leukocyte-endothelial interactions. *Blood* 1985; **65**: 513–525.
17. Anderson DC, Schmalsteig FC, Finegold MJ *et al.* The severe and moderate phenotypes of heritable Mac-1, LFA-1 deficiency: their quantitative definition and relation to leukocyte dysfunction and clinical features. *J Inf Dis* 1985; **152**: 668–689.
18. Duling BR. The preparation and use of the hamster cheek pouch for studies of the microcirculation. *Microvasc Res* 1973; **5**: 423–429.
19. Abbassi O, Lane CL, Krater S, *et al.* Canine neutrophil margination mediated by lectin adhesion molecule-1 *in vitro*. *J Immunol* 1991; **147**: 2107–2115.
20. Goetzl EJ, Pickett WC. Novel structural determinants of the human neutrophil chemotactic activity of leukotriene $B_4$. *J Exp Med* 1981; **153**: 482–487.
21. Smith MJH. Biological activities of leukotriene $B_4$ (isomer III). In: Samuelsson B, Paoletti R, eds. *Leukotrienes and other lipoxygenase products*. New York: Raven Press, 1982; 283–292.
22. Feinmark SJ, Lindgren JA, Claesson HE, Malmsen C, Samuelsson B. Stimulation of human leukocyte degranulation by leukotriene $B_4$ and its oxidized metabolites. *FEBS Lett* 1981; **136**: 141–144.
23. Hafstrom I, Palmblad J, Malmsen CL, Radmark O, Samuelsson B. Leukotriene $B_4$: A stereospecific stimulator for release of lysosomal enzymes from neutrophils. *FEBS Lett* 1981; **130**: 146–148.
24. Rae SA, Smith MJH. The stimulation of lysosomal enzyme secretion from human polymorphonuclear leucocytes by leukotriene $B_4$. *J Pharm Pharmacol* 1981; **33**: 616–617.
25. Showell HJ, Nacache PH, Borgeat R, *et al.* Characterization of the secretory activity of leukotriene $B_4$ toward rabbit neutrophils. *J Immunol* 1982; **128**: 811–816.
26. House SD, Lipowsky HH. Leukocyte-endothelium adhesion: Microhemodynamics in mesentery of the cat. *Microvasc Res* 1987; **34**: 363–379.
27. Gallin JI, Seligmann BE. Mobilisation and adaptation of human neutrophil chemoattractant fMet–Leu–Phe–receptors. *Fed Proc* 1984; **43**: 2732–2736.
28. Hoover RL, Karnosovsky MJ, Austen KF, Corey EJ, Lewis RA. Leukotriene $B_4$ action on endothelium mediates augmented neutrophil/endothelial adhesion. *Proc Natl Acad Sci USA* 1984; **81**: 2191–2193.
29. Marchesi VT, Florey HW. Electron micrographic observations on the migration of leucocytes. *Q J Exp Physiol* 1960; **45**: 343–348.
30. Katori M, Oda T, Nagai K. A site of action of dexamethasone on leukocyte extravasation in microcirculation. *Agents & Actions* 1990; **29**: 24–26.
31. Oda T, Katori M. Inhibition site of dexamethasone on extravasation of polymorphonuclear leukocytes in hamster cheek pouch microcirculation. *J Leukocyte Biol* 1992; **52**: 339–342.

ACKNOWLEDGEMENTS. We wish to thank Mrs C. Shima, Mrs M. Takagi and Miss M. Takahara for their technical assistance. We also are obliged to Mr O. Katsumata for his skilful technical assistance in electron microscopy. This study was partly supported by a Grant-in-aid from the Ministry of Education, Science and Culture (#01770137) and by the Uehara Memorial Foundation.

**Received 14 September 1992; accepted 24 September 1992**
